# A Rare Cause of Testicular Metastasis: Upper Tract Urothelial Carcinoma

**DOI:** 10.1155/2014/759858

**Published:** 2014-07-13

**Authors:** Alper Nesip Manav, Ercan Kazan, Mehmet Şirin Ertek, Akın Soner Amasyalı, Nil Çulhacı, Haluk Erol

**Affiliations:** ^1^Department of Urology, School of Medicine, Adnan Menderes University, 09100 Aydın, Turkey; ^2^Urology Clinic, Batman Kozluk State Hospital, Kozluk, 72400 Batman, Turkey; ^3^Department of Pathology, School of Medicine, Adnan Menderes University, 09100 Aydın, Turkey

## Abstract

Metastatic testicular cancers are rare. Primary tumor sources are prostate, lung, and gastrointestinal tract for metastatic testicular cancers. Metastasis of urothelial carcinoma (UC) to the testis is extremely rare. Two-thirds of upper tract urothelial carcinoma (UTUC) is of invasive stage at diagnosis and metastatic sites are the pelvic lymph nodes, liver, lung, and bone. We report a rare case of metastatic UTUC to the testis which has not been reported before, except one case in the literature. Testicular metastasis of UC should be considered in patients with hematuria and testicular swelling.

## 1. Introduction

Upper tract urothelial carcinomas (UTUC) are rare, with an incidence of 1–3/100,000 in western countries [1, 2]. UTUC constitutes 5–10% of all urothelial carcinomas (UC). Additionally, although not a diagnostic symptom, micro- or macrohematuria may be observed. UTUC has a peak incidence at seventy to eighty years of age. Sixty percent of UTUCs are invasive at diagnosis, and systemic evaluation must be performed carefully [2, 3].

Metastases to the testis are rare, and such cancers are determined incidentally in orchiectomy or autopsy specimens [4]. The most common primary tumor sources include prostate, lung, and gastrointestinal tract tumors, although melanoma may be a less common primary tumor source [4, 5]. In this report, we present a rare case of testicular metastasis of UTUC that was determined incidentally and review the literature.

## 2. Case Report

**Figure 1:**
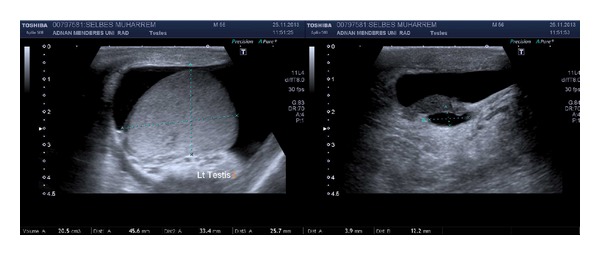
Ultrasonographic images revealed hydrocele and epididymal cyst in the left hemiscrotum.

**Figure 2:**
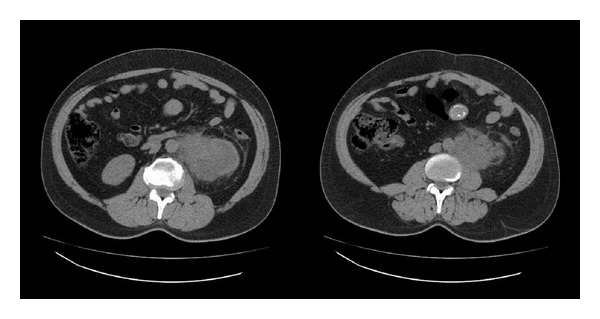
8.5 × 7.5 cm lymph node surrounding the left proximal ureter and the abdominal aorta in the left para-aortic area on noncontrast computed tomography.

A fifty-six-year-old man presented with intermittent left flank pain, swelling of the left testis, and painless intermittent macroscopic hematuria. He had no other chronic diseases and did not smoke. The left testis presented a diffuse, tough, irregular, and minimal hydrocele in the left hemiscrotum upon physical examination. Microscopic hematuria was determined in a urine analysis. The plasma creatinine level was normal, and testicular tumor markers were as follows: lactate dehydrogenase of 229 U/L (N: 125–243), alpha fetoprotein of 2.52 ng/mL (N: 0–13.4), and *β* human chorionic gonadotropin < 1.2 mIU/mL (N: 0–5). Ultrasonography (USG) was immediately performed to determine the presence of testicular cancer; both testes appeared normal except for a hydrocele in the left hemiscrotum (Figure 1). We identified grade 3 hydronephrosis in the left kidney, and the left renal parenchyma was thinner than that of the right. There was an 8.5 × 7.5 cm lymph node surrounding the left proximal ureter and the abdominal aorta in the left para-aortic area on noncontrast computed tomography (CT), which was performed because of hematuria and flank pain (Figure 2). The radiology department reported metastatic testicular cancer. A few pathological hyperdense areas were observed on a chest radiograph.

**Figure 3:**
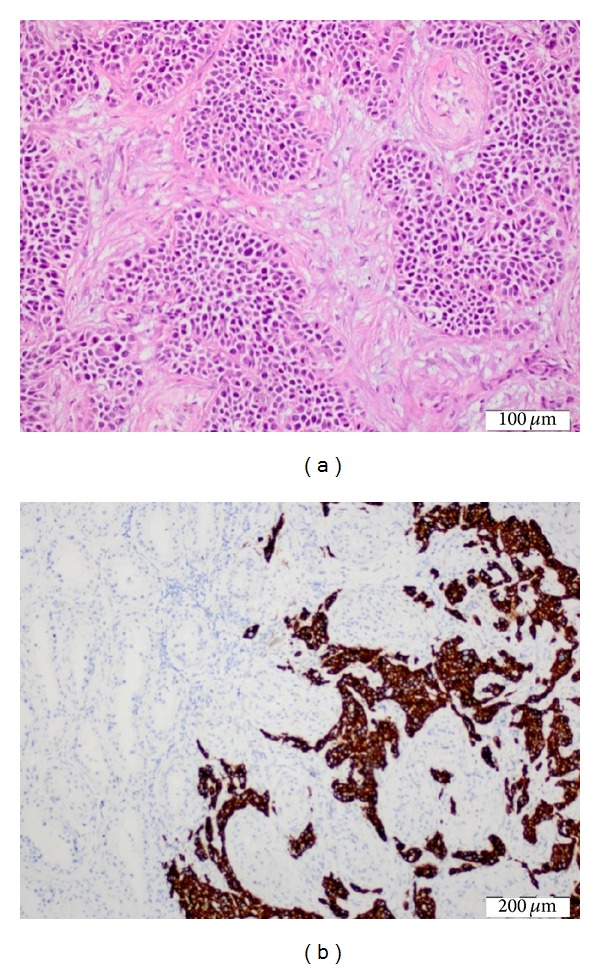
Histopathologic findings of UC metastasis to the left testis ((a) hematoxylin and eosin x20 and (b) cytokeratin x10).

Cystoscopy revealed a normal bladder and an obstructed left proximal ureter on retrograde pyelography (RGP). The contrast agent could not pass through mid-ureter to proximal segment at RGP. But we could not make differential diagnosis external compression or intraureteral mass for cause of hydronephrosis. Bladder and left ureteral cytology was benign. But we know that urine cytology has low sensitivity for UTUC even for high-grade lesions. After endoscopic investigation, we performed left radical orchiectomy and made a preliminary diagnosis of testicular cancer perioperatively. The pathological result of the left testis was a 3.8 cm malignant epithelial tumor metastasis in the paratesticular area. The tumor included multiple solid areas and lymphovascular, fat, and muscle tissue invasion. The pathology department identified the tumor as the UC metastasis to the left testis (Figure 3). Metastases on the pelvic bone and left sixth rib were revealed by total bone scintigraphy. We thought metastatic UTUC through pathological report, lymph nodes in CT, scintigraphy, and RGP. We planned a systemic gemcitabine-cisplatin chemotherapy regimen.

## 3. Discussion

In two-thirds of cases, UC is of invasive grade at diagnosis [2, 3]. The most common metastasis of UC is to the regional lymph nodes, although metastasis to the bone, lung, and liver is not rare [6]. By contrast, metastasis to the testis is very rare. A few case reports describing UC metastasis to the testis are available in the literature. All of the cases in those reports of metastatic UC to the testis had tumor origins in the bladder or prostatic urethra [7, 8]. Only one case report is available in the literature regarding UTUC metastasizing to the testis [9]. Some authors estimate that testicular metastasis of UC can occur through the ejaculatory ducts [7, 10].

Cancer metastasis to the testis is very rare, and such cancers are identified incidentally in 2–4% of orchiectomy specimens [4]. One study performed on autopsy specimens has shown that secondary testicular cancers are found with an incidence of 0.1%; the most common origins were prostate, lung, and gastrointestinal tumors and melanoma [5].

Swelling of the left testis and left para-aortic lymph node on CT is suggestive of testicular cancer. We performed cystoscopy and cytological examination of the bladder and separated the left ureter to exclude bladder UC and UTUC. The most important oversight in this case was the lack of ureterorenoscopy to exclude UTUC with potential testicular metastasis.

## 4. Results

Metastatic testicular tumors are observed only very rarely. Our case is the second case of testicular metastasis of UTUC reported in the literature. The most common pathway of tumor spread in UC is the lymphovascular system, and we hypothesize that malignant cells can metastasize through the lymphovascular system after lymph node invasion or through reverse blood flow in the left testicular vein, which can be caused by varicocele formation after renal or testicular vein invasion. Testicular metastasis of UC should be considered in patients with hematuria and testicular swelling.
